# Preclinical Models of Colorectal Cancer Liver Metastasis: Therapeutic Evaluation and Translational Implications

**DOI:** 10.32604/or.2026.079556

**Published:** 2026-06-16

**Authors:** Ye Ri Han, Sang Bong Lee

**Affiliations:** 1 Department of Chemistry, Duksung Women’s University, Seoul, Republic of Korea; 2 Department of Life Science, Dongguk University, Dongguk-ro, Ilsandong-gu, Goyang-si, Gyeonggi-do, Republic of Korea; 3 Department of Biomedical Sciences, Chonnam National University Medical School 264, Hwasun-eup, Hwasun-gun, Jeollanam-do, Republic of Korea; 4 SimVista Inc., A-13, 194-25 Osongsaengmueong1-ro, Osong-eup, Heungdeok-gu, Cheongju-si, Chungcheongbuk-do, Republic of Korea

**Keywords:** Colorectal cancer liver metastasis (CRLM), orthotopic tumor models, liver-directed therapy, tumor microenvironment, immunotherapy, translational research

## Abstract

Colorectal cancer liver metastasis (CRLM) remains a leading cause of cancer-related mortality, with clinical outcomes limited by biological heterogeneity and inconsistent therapeutic responses. Despite advances in systemic chemotherapy, targeted agents, immunotherapy, and liver-directed interventions, the translation of preclinical efficacy into clinical benefit remains suboptimal, highlighting the need for predictive experimental models. However, therapeutic efficacy in CRLM is highly model-dependent, and discrepancies between preclinical findings and clinical outcomes often arise from differences in biological fidelity across experimental platforms. This review critically examines preclinical platforms used to study CRLM, with emphasis on orthotopic and metastatic models that recapitulate hepatic colonization, tumor–microenvironment interactions, and immune regulation. We evaluate methodological innovations that enhance anatomical fidelity and reproducibility, including tissue adhesive–based implantation and biomaterial-assisted strategies. Importantly, we analyze how different models influence therapeutic assessment across systemic, immune-based, metabolic, and liver-directed treatments, and discuss their ability to predict clinical responses. By integrating insights from experimental studies with key clinical evidence, we delineate the strengths and limitations of current platforms and propose principles for rational model selection to improve translational success in CRLM research.

## 1 Introduction

Colorectal cancer (CRC) remains a leading cause of cancer-related mortality worldwide, with liver metastasis constituting the most common and clinically consequential site of distant spread [1,2]. The development of colorectal cancer liver metastasis (CRLM) is a multistep process that encompasses tumor cell dissemination, hepatic colonization, and adaptation to the unique immunological and stromal environment of the liver [3,4]. As a result, therapeutic responses in CRLM are highly heterogeneous, reflecting both tumor-intrinsic features and microenvironmental influences [5]. These challenges underscore the need for experimental systems that faithfully recapitulate the metastatic cascade and enable reliable evaluation of therapeutic efficacy. Recent advances in preclinical modeling have addressed these limitations by improving tumor localization, metastatic reproducibility, and biological relevance. In particular, orthotopic implantation strategies incorporating tissue adhesive–based techniques have enabled the generation of reproducible metastatic CRC models with consistent liver involvement, thereby providing robust platforms for translational oncology research [3].

Using such metastatic platforms, a diverse array of therapeutic strategies has been investigated in preclinical settings. Combination approaches integrating personalized immunotherapeutic modalities with molecular targeted agents have demonstrated the capacity to remodel the immune landscape of microsatellite-stable CRLM, a population that is largely refractory to immune checkpoint blockade [6]. In parallel, biomimetic nanomedicine systems have been developed to enhance intra-tumoral drug delivery, eradicate tumor-associated microbial components, and potentiate the efficacy of conventional chemotherapy within hepatic metastases [7]. Localized drug delivery strategies, including thermosensitive and drug-eluting hydrogel systems, have further expanded the therapeutic repertoire by enabling sustained intra-tumoral exposure and improved control of both orthotopic primary tumors and liver metastatic lesions [8,9].

Immunomodulatory strategies have been intensively explored in metastatic CRC models. Engineered nanodrugs and cell membrane–modified delivery platforms have been shown to sensitize immune-desert liver metastases to immune checkpoint inhibition [10], while nucleic acid–based nanotherapeutics targeting metastasis-associated signaling pathways have effectively suppressed hepatic metastatic burden [11]. Beyond adaptive immunity, activation of innate immune pathways—particularly macrophage STING signaling and natural killer cell co-stimulation—has emerged as a critical mechanism for constraining metastatic progression in the liver [12]. Multifunctional nanoplatforms combining physical modalities, such as sonodynamic therapy, with immune modulation further highlight the therapeutic potential of rational combinatorial strategies designed to overcome immune resistance within the hepatic metastatic niche [13].

At the molecular level, accumulating evidence has elucidated signaling pathways and microenvironmental factors that govern metastatic outgrowth and therapeutic responsiveness. Genetic regulators that modulate sensitivity to cytotoxic chemotherapy [14], metabolic and inflammatory conditions such as non-alcoholic fatty liver disease that promote immune evasion [4], and metabolic interventions capable of reversing immune checkpoint resistance [15,16] collectively illustrate the biological complexity of CRLM. In addition, naturally derived compounds and phytochemicals have demonstrated antimetastatic activity through reprogramming of the hepatic immune milieu and suppression of stromal cell activation [17,18]. Intercellular communication mediated by extracellular vesicles, exosomes, and microRNAs further contributes to metastatic niche formation and represents an emerging axis for therapeutic intervention [19].

Increasing attention has also been directed toward the role of the gut–liver axis in regulating metastatic progression. Modulation of the intestinal microbiome through probiotic supplementation or non-absorbable antibiotic treatment has been shown to alter bile acid metabolism and immune responses, resulting in reduced liver metastatic burden in experimental models [20,21]. Traditional and herbal medicines have similarly provided mechanistic insight into stromal–tumor crosstalk by targeting extracellular vesicle–mediated fibroblast activation and hepatic microenvironmental remodeling [22,23]. Moreover, therapeutic strategies aimed at immunosuppressive myeloid populations, including macrophages and myeloid-derived suppressor cells, have demonstrated the feasibility of restoring antitumor immunity and enhancing responsiveness to immune checkpoint blockade in CRLM [5].

Local and liver-directed therapies represent another critical dimension of CRLM management and have been extensively evaluated in preclinical studies. Combinatorial strategies integrating microwave or radiofrequency ablation with immune checkpoint inhibitors or nano vaccine-based adjuvant therapies have yielded synergistic antitumor effects, induced durable immune memory and suppressed metastatic recurrence [24,25].

Efforts to remodel the hepatic immune microenvironment and dismantle T-cell exclusion niches have reinforced the concept that immune contexture is a central determinant of therapeutic success in liver metastasis [26]. Furthermore, targeting stromal and perivascular components, including pericytes and cancer-associated fibroblasts, has revealed previously underappreciated mechanisms driving metastatic colonization, immune suppression, and therapeutic resistance [27,28]. Additional approaches exploiting regulated cell death pathways, antibody-based targeting strategies, and nanoparticle-mediated immune activation continue to broaden the preclinical therapeutic landscape [29,30,31].

The translational relevance of these experimental advances is underscored by parallel developments in the clinical management of CRLM. Post hoc analyses and randomized clinical trials have demonstrated that optimized systemic regimens can facilitate conversion to respectability and improve survival outcomes in selected patient populations [32,33]. Surgical resection combined with adjuvant or perioperative chemotherapy remains the cornerstone of curative-intent treatment; however, recurrence rates remain substantial, emphasizing the need for improved patient stratification and adjunctive therapeutic strategies [34,35]. Regional treatment approaches, including hepatic arterial chemotherapy, intra-arterial targeted delivery, and gene-based immunotherapy combinations, have further diversified therapeutic options for liver-dominant disease [36,37]. Additional liver-directed modalities such as high-intensity focused ultrasound and yttrium-90 radioembolization have highlighted the importance of intrahepatic disease control and immune modulation in advanced CRLM [38,39].

Advances in response assessment and precision oncology have complemented therapeutic innovation. Radiomics-based prediction models and molecular imaging approaches now provide noninvasive tools for assessing treatment response, tumor heterogeneity, and clinical outcomes following liver-directed therapies [40,41]. Landmark randomized trials have established perioperative chemotherapy [2,42] and conversion strategies as standards of care for both resectable and initially unresectable CRLM, forming the foundation of contemporary clinical guidelines [43,44,45]. More recently, immuno–positron emission tomography using site-specifically labeled tracers has enabled *in vivo* assessment of target expression heterogeneity, offering a conceptual bridge between preclinical modeling and patient-specific therapeutic decision-making [46].

In this review, we do not aim to provide formal evidence grading or systematic meta-analytic comparison of therapeutic efficacy across models. Rather, our primary objective is to critically examine how the choice of preclinical platform influences the interpretation of therapeutic response and the perceived translational potential of emerging strategies in colorectal cancer liver metastasis (CRLM).

Importantly, this review is intentionally structured around a translational evaluation paradigm rather than a descriptive summary of therapeutic modalities. Our central thesis is that therapeutic efficacy in CRLM is fundamentally model-contingent, and that failure to account for model-dependent biological constraints has contributed to historical translational discrepancies between preclinical findings and clinical outcomes.

Specifically, we examine how model-dependent immune context, metastatic synchronization, and stromal representation influence discrepancies between radiographic response, immune activation, and long-term survival observed in clinical trials.

Accordingly, we operationalize an explicit evaluative framework defining preclinical model relevance across five analytical dimensions: (i) anatomical fidelity of metastatic dissemination, (ii) immune competence and hepatic microenvironment representation, (iii) reproducibility and scalability, (iv) alignment with established clinical biological behavior, and (v) relevance to clinically meaningful endpoints such as progression-free survival, overall survival, and conversion-to-resection rates. This framework serves as the analytical foundation through which therapeutic evidence is interpreted throughout the manuscript.

Within this review, the term “biologically relevant” therefore refers to models that reproduce not only anatomical tumor localization but also immune competence, hepatic microenvironmental context, and clinically aligned response patterns.

**Figure 1:**
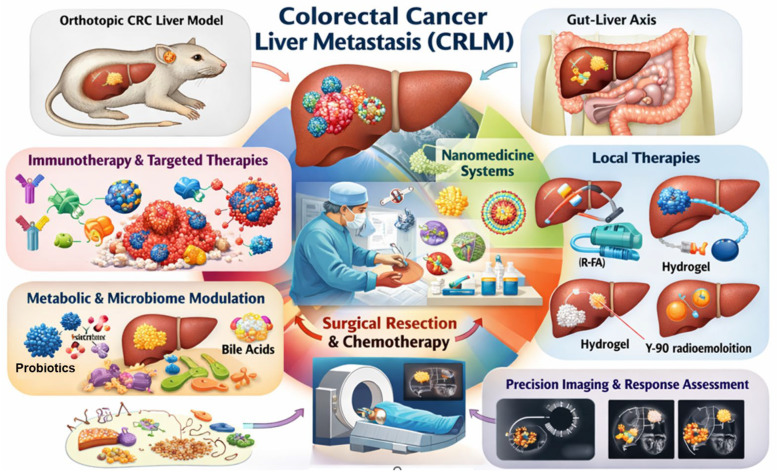
**Integrated landscape of preclinical modeling and therapeutic strategies for colorectal cancer liver metastasis (CRLM).** This schematic illustrates the biological and therapeutic framework of colorectal cancer liver metastasis (CRLM) as discussed in the Introduction. At the center, CRLM is depicted as a complex disease driven by interactions between metastatic tumor cells and the hepatic microenvironment. Orthotopic and metastatic preclinical models, including liver-targeted implantation approaches, are shown as foundational platforms for investigating metastatic progression and therapeutic response. Surrounding panels highlight major therapeutic and biological axes evaluated in these models, including immunotherapy and molecular targeted therapies, nanomedicine-based delivery systems, and local liver-directed interventions such as radiofrequency ablation, hydrogel-mediated drug delivery, and yttrium-90 radioembolization. Additional layers emphasize the contribution of metabolic regulation and the gut–liver axis, including microbiome-derived factors and bile acid signaling, to metastatic colonization and immune modulation. Surgical resection and systemic chemotherapy remain central components of curative-intent treatment, while advanced imaging modalities enable precision assessment of tumor burden, therapeutic response, and intra-tumoral heterogeneity. Together, this figure summarizes how biologically relevant preclinical models integrate diverse therapeutic strategies and translational endpoints to inform the clinical management of CRLM. The figure was created using BioRender (BioRender Inc., Toronto, ON, Canada) and assembled in Microsoft PowerPoint (Microsoft 365, Microsoft Corporation, Redmond, WA, USA).

By categorizing experimental systems according to anatomical fidelity, immune competence, microenvironmental representation, and reproducibility, we seek to clarify how model-dependent bias may contribute to discrepancies between preclinical promise and clinical outcomes. Through this lens, the review emphasizes rational model selection as a central determinant of translational relevance, rather than positioning preclinical findings as direct predictors of clinical efficacy (Fig. 1). To operationalize this objective, we propose a structured evaluative framework through which CRLM preclinical models can be critically interpreted. Importantly, this framework is intended not only as a descriptive categorization of model characteristics but as a translational bridge linking experimental design to clinical trial architecture. By explicitly mapping model-specific biological features to clinical endpoints and therapeutic strategies, we aim to clarify how preclinical evidence should inform trial design, patient selection, and endpoint prioritization in CRLM. Specifically, we examine models across five translational axes: (i) anatomical fidelity of metastatic dissemination, (ii) immune competence and hepatic microenvironment representation, (iii) reproducibility and experimental scalability, (iv) alignment of therapeutic response patterns with known clinical biology, and (v) relevance to clinically meaningful endpoints such as progression-free survival, overall survival, and conversion-to-resection rates. By defining these criteria, the present review moves beyond descriptive categorization and instead provides an analytical lens for assessing how model-dependent bias may shape therapeutic interpretation.

To ensure comprehensive coverage, relevant literature was identified through systematic searches of PubMed, Web of Science, and Scopus databases. Articles published between January 2000 and March 2025 were screened using combinations of the keywords “colorectal cancer liver metastasis”, “CRLM”, “orthotopic model”, “metastatic model”, “immunotherapy”, “nanomedicine”, “liver-directed therapy”, and “translational research”. Both preclinical and clinical studies were included if they provided mechanistic insight or translational relevance to CRLM. Case reports, non-peer-reviewed materials, and studies lacking methodological clarity were excluded. Reference lists of key articles were additionally reviewed to ensure completeness.

## 2 Preclinical Models of Colorectal Cancer Liver Metastasis

### 2.1 Limitations of Conventional Subcutaneous and Ectopic Models

Conventional subcutaneous tumor models have historically been employed for anticancer drug evaluation owing to their technical simplicity and ease of tumor monitoring. However, these ectopic models fail to recapitulate the complex biological processes underlying colorectal cancer liver metastasis (CRLM). In particular, subcutaneous implantation does not reproduce the multistep metastatic cascade—including intravasation, hepatic arrest, extravasation, and colonization within the liver parenchyma—which critically shapes metastatic fitness and therapeutic vulnerability. As a consequence, key determinants of metastatic progression and treatment response are fundamentally absent in ectopic settings.

The liver represents a highly specialized immunological organ characterized by immune tolerance, resident macrophages (Kupffer cells), hepatic stellate cells, sinusoidal endothelial cells, and unique metabolic and vascular architectures. Nevertheless, standard murine systems do not fully reproduce the chronic fibrotic remodeling, chemotherapy-induced sinusoidal injury, or long-standing immune exhaustion frequently observed in human CRLM, and these cross-species differences should be considered when interpreting therapeutic magnitude. These liver-specific components collectively establish a permissive yet tightly regulated metastatic niche that governs tumor cell survival, immune evasion, and therapeutic resistance. Subcutaneous models lack these hepatic microenvironmental cues and therefore inadequately reflect critical features of CRLM biology, including stromal activation, myeloid cell–mediated immune suppression, and therapy-induced remodeling of the tumor microenvironment [3,4,5].

The translational limitations of ectopic models have become particularly evident in the context of targeted and immune-based therapies. Despite robust preclinical efficacy, several therapeutic strategies have demonstrated limited or inconsistent benefit in clinical trials involving CRLM patients, underscoring the disconnect between ectopic tumor models and liver-dominant metastatic disease [42,44,45]. These discrepancies suggest that therapeutic responses observed in subcutaneous models may overestimate clinical benefit by failing to account for liver-specific immune regulation and stromal interactions.

Importantly, this overestimation is not merely theoretical but is reflected in historical translational failures. Several targeted and immune-based strategies demonstrating robust tumor regression in ectopic settings have failed to produce comparable survival benefit in CRLM clinical trials. One major explanatory factor is the absence of hepatic immune tolerance mechanisms—such as Kupffer cell–mediated immunoregulation, sinusoidal endothelial modulation, and myeloid-derived suppressor cell recruitment—which fundamentally reshape therapeutic responsiveness within the liver. Consequently, ectopic tumor regression should not be interpreted as predictive of metastatic efficacy without accounting for organ-specific immune constraints.

**Figure 2:**
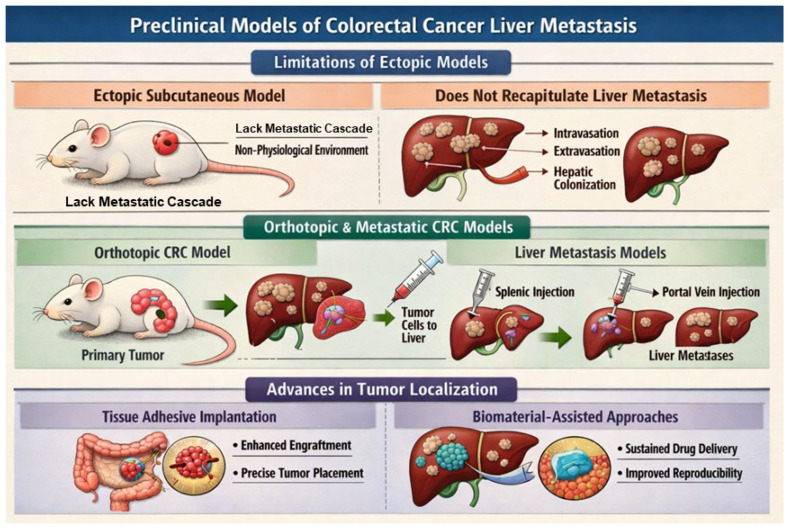
**Preclinical models of colorectal cancer liver metastasis and advances in tumor localization.** This figure summarizes the major preclinical modeling strategies used to study colorectal cancer liver metastasis (CRLM) and highlights key methodological advances that improve biological relevance and reproducibility. The upper panel illustrates the limitations of conventional ectopic subcutaneous tumor models, which fail to recapitulate the metastatic cascade and do not reproduce critical steps of liver metastasis, including intravasation, extravasation, and hepatic colonization. The absence of the liver-specific microenvironment in ectopic models limits their ability to accurately predict therapeutic responses in CRLM. The middle panel depicts orthotopic and metastatic CRC models, including orthotopic implantation of primary colorectal tumors and liver metastasis models generated via splenic or portal vein injection. These approaches enable physiologically relevant tumor dissemination and allow focused investigation of hepatic colonization and metastatic growth within the liver microenvironment. The lower panel highlights recent advances in tumor localization and model reproducibility. Tissue adhesive–based implantation strategies enhance tumor engraftment and enable precise spatial placement of orthotopic tumors, while biomaterial-assisted approaches, such as hydrogel-based systems, support sustained drug delivery and improve experimental consistency. Collectively, these modeling platforms provide robust and translationally relevant tools for evaluating therapeutic efficacy and microenvironment-dependent treatment responses in colorectal cancer liver metastasis. The figure was created using BioRender (BioRender Inc., Toronto, ON, Canada) and assembled in Microsoft PowerPoint (Microsoft 365, Microsoft Corporation, Redmond, WA, USA).

Collectively, these limitations highlight the inadequacy of conventional subcutaneous and ectopic tumor models for studying CRLM and reinforce the need for anatomically and biologically relevant metastatic models that faithfully recapitulate the hepatic microenvironment. This recognition has driven the development of orthotopic and liver-targeted metastatic models, which are increasingly regarded as essential platforms for predictive therapeutic evaluation (Fig. 2 upper panel).

### 2.2 Orthotopic and Metastatic CRC Models

To overcome the limitations of ectopic implantation, orthotopic and metastatic CRC models have been developed to better recapitulate the natural history of CRLM [3,8]. Orthotopic implantation of colorectal cancer cells or tumor fragments into the cecum or colon enables spontaneous dissemination to the liver through physiologically relevant routes, closely mimicking clinical patterns of metastasis [3]. These models preserve tumor–stromal interactions at the primary site while allowing subsequent evaluation of hepatic colonization and metastatic outgrowth [3,8].

Among metastatic approaches, splenic or portal vein injection models are widely used to generate reproducible liver metastases with high efficiency. In these systems, tumor cells introduced into the splenic circulation rapidly seed the liver via the portal vein, allowing focused investigation of hepatic colonization, metastatic growth, and treatment response within the liver microenvironment [7,20]. Although these models bypass early steps of metastasis, such as primary tumor invasion, they provide robust and temporally controlled platforms for evaluating liver-specific therapeutic effects.

It is important to recognize that most cell line–derived models incompletely recapitulate the intratumoral heterogeneity and long-term clonal evolution characteristic of human CRLM, potentially limiting their ability to model immune editing and therapy-driven subclonal selection [5,26].

However, the controlled efficiency of splenic or portal vein injection models may introduce a distinct form of translational bias. By artificially synchronizing metastatic seeding, these systems often generate homogeneous tumor burdens that differ from the temporal and clonal heterogeneity observed in patients. As a result, therapeutic efficacy assessed under these synchronized conditions may underestimate the impact of spatial heterogeneity and immune editing processes that occur during natural metastatic evolution [11,23].

Recent studies have demonstrated the utility of these models in assessing diverse therapeutic strategies, including local drug delivery systems, nanomedicine-based therapies, and immune-modulating interventions. Orthotopic CRC models incorporating liver metastasis have been successfully applied to evaluate thermosensitive hydrogel-mediated chemotherapy and local tumor control [8], while splenic injection models have enabled mechanistic studies of nanoparticle-mediated gene delivery and microbiome-driven modulation of metastatic burden [11,23]. Importantly, orthotopic and metastatic models have emerged as indispensable tools for interrogating immune responses, stromal remodeling, and treatment-induced changes within the hepatic metastatic niche [1] (Fig. 2 middle panel).

### 2.3 Methodological Advances to Improve Reproducibility and Tumor Localization

Despite their biological relevance, orthotopic and metastatic CRC models have historically suffered from variability in tumor engraftment, inconsistent metastatic burden, and technical challenges associated with surgical implantation. To address these limitations, recent methodological advances have focused on improving tumor localization, engraftment efficiency, and experimental reproducibility.

Importantly, these refinements reduce inter-animal variability and enhance the reliability of therapeutic response interpretation, thereby strengthening the translational validity of model-based findings.

One notable innovation is the development of tissue adhesive–based orthotopic implantation techniques. By using biological adhesives to anchor tumor fragments or cell suspensions at the implantation site, these approaches minimize cell leakage, reduce off-target tumor formation, and enable precise spatial control of tumor growth. This strategy has been shown to significantly enhance engraftment consistency and metastatic reproducibility, thereby improving the reliability of therapeutic evaluation in orthotopic CRC liver metastasis models [3].

In parallel, biomaterial-assisted strategies—such as injectable hydrogels and drug-eluting matrices—have been employed not only as therapeutic platforms but also as tools for tumor localization and sustained drug delivery. These biomaterials facilitate controlled release of anticancer agents within orthotopic or metastatic lesions and allow investigation of local treatment effects within the hepatic microenvironment [8,9]. By stabilizing tumor architecture and reducing inter-animal variability, biomaterial-assisted approaches contribute to more reproducible assessment of therapeutic efficacy and immune modulation.

Collectively, these methodological refinements have transformed orthotopic and metastatic CRC models from technically challenging systems into robust and scalable platforms for translational oncology research. Their integration into preclinical pipelines is essential for improving the predictive value of experimental studies and bridging the gap between preclinical findings and clinical outcomes in CRLM. (Fig. 2 lower panel) The key characteristics, advantages, and limitations of commonly used preclinical CRLM models are summarized in Table 1.

**Table 1:** Preclinical models for colorectal cancer liver metastasis: characteristics, advantages, and limitations.

Model Type	Implantation Method	Metastatic Features	Advantages	Limitations	Key References
**Subcutaneous**	Flank injection	None	Simple, high throughput	Lacks hepatic TME, poor predictability	—
**Orthotopic CRC**	Cecal/colonic implantation	Spontaneous liver mets	Physiologic dissemination	Technical variability	[3,8]
**Splenic injection**	Splenic/portal injection	Rapid liver colonization	High reproducibility	Bypasses early metastasis	[11,23]
**Adhesive-based orthotopic**	Orthotopic + tissue adhesive	Controlled local & liver mets	High localization, reproducible	Surgical skill required	[3]
**Biomaterial-assisted**	Hydrogel-assisted implantation	Localized growth	Sustained delivery, reduced leakage	Model-specific optimization	[8,9]

**Note:** CRC, colorectal cancer; CRLM, colorectal cancer liver metastasis; TME, tumor microenvironment. “—” indicates that no specific key reference is assigned or that the feature is not applicable to the indicated model.

## 3 Therapeutic Evaluation Using CRLM Preclinical Models

**Figure 3:**
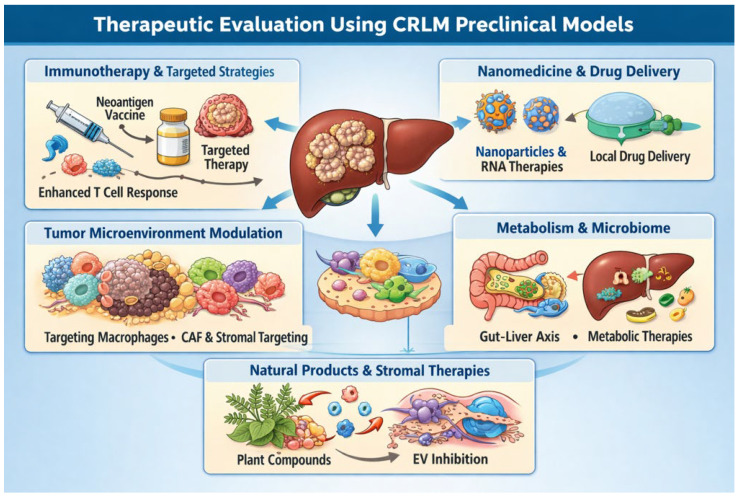
**Therapeutic evaluation strategies using preclinical models of colorectal cancer liver metastasis (CRLM).** This schematic illustrates the major therapeutic strategies evaluated using preclinical models of colorectal cancer liver metastasis (CRLM). At the center, liver metastases are depicted as dynamic lesions shaped by interactions between tumor cells and the hepatic microenvironment. Surrounding panels summarize key therapeutic axes investigated in biologically relevant metastatic models. Immunotherapy and targeted strategies include personalized neoantigen vaccination and molecular targeted agents designed to enhance antitumor T-cell responses and overcome resistance to immune checkpoint blockade. Nanomedicine and drug delivery approaches encompass biomimetic nanoparticles, RNA-based therapeutics, and local delivery systems that improve drug accumulation and therapeutic efficacy within hepatic metastases. Tumor microenvironment modulation highlights interventions targeting immunosuppressive macrophages, cancer-associated fibroblasts, and stromal components to restore antitumor immunity. Metabolic and microbiome-focused therapies emphasize the role of the gut–liver axis, bile acid signaling, and metabolic regulation in shaping metastatic progression and treatment responsiveness. Natural products and stromal-targeted therapies include phytochemicals and strategies aimed at disrupting extracellular vesicle–mediated tumor–stroma communication. Together, this figure summarizes how diverse therapeutic modalities are integrated within CRLM preclinical models to evaluate treatment efficacy, immune modulation, and microenvironment-dependent therapeutic responses. The figure was created using BioRender (BioRender Inc., Toronto, ON, Canada) and assembled in Microsoft PowerPoint (Microsoft 365, Microsoft Corporation, Redmond, WA, USA).

Preclinical models of colorectal cancer liver metastasis (CRLM) have become indispensable platforms for evaluating therapeutic strategies that target not only tumor-intrinsic vulnerabilities but also the hepatic metastatic microenvironment. Importantly, the therapeutic outcomes discussed in this section should be interpreted within the biological and technical constraints of the respective experimental models. Rather than ranking therapeutic efficacy, we focus on how model-specific features—such as immune competence, hepatic microenvironment fidelity, and metastatic reproducibility—shape observed treatment responses and influenced translational expectations. Importantly, the therapeutic strategies discussed herein span a spectrum from exploratory mechanistic investigations to approaches with emerging clinical correlates. While many studies demonstrate compelling biological effects within controlled experimental settings, not all interventions currently possess equivalent levels of translational maturity. In this review, we explicitly distinguish mechanistic proof-of-concept findings from strategies that have demonstrated reproducibility across complementary models or that align with ongoing or completed clinical investigations. Such differentiation is essential to avoid overinterpretation of preclinical efficacy as immediate clinical readiness. In the context of CRLM, therapeutic evaluation should extend beyond the magnitude of tumor shrinkage. A structured assessment requires consideration of (i) reproducibility across independent models or laboratories, (ii) comparative efficacy relative to established or competing strategies, (iii) dose–response relationships and therapeutic windows, and (iv) durability of response, including recurrence and survival endpoints. Where available, attenuated or negative findings should also inform interpretation, as these often reveal model-specific limitations that shape translational reliability. Importantly, therapeutic outcomes observed in these models should be interpreted in the context of their translational intent. Immune-competent orthotopic models are particularly suited to inform immunotherapy combination trial design, whereas highly synchronized splenic injection models may better model early hepatic colonization but require caution when extrapolating durability of response. Thus, model choice should be aligned with the specific clinical question being addressed. The biological complexity of CRLM—shaped by immune tolerance, stromal interactions, metabolic regulation, and microbiome-derived factors—necessitates integrative therapeutic approaches that extend beyond conventional cytotoxic regimens. Recent studies employing biologically relevant metastatic models have enabled systematic interrogation of immunotherapy, targeted agents, nanomedicine-based delivery systems, and microenvironment-focused interventions, providing critical insights into model-dependent therapeutic responses. (Fig. 3) A comparative overview of therapeutic strategies evaluated across preclinical CRLM models is presented in Table 2.

All therapeutic findings discussed in this section are interpreted through the predefined five-axis translational framework introduced in the Introduction, ensuring analytical consistency and preventing descriptive overinterpretation of model-specific efficacy signals.

**Table 2:** Therapeutic strategies evaluated in preclinical models of colorectal cancer liver metastasis.

Therapeutic Category	Representative Approaches	Targeted Mechanisms	Model Type	Key Outcomes	References
**Immunotherapy & targeted combinations**	Neoantigen vaccine + TKI, anti–PD-1/PD-L1 sensitization	T-cell activation, macrophage reprogramming	Orthotopic, liver mets	Tumor regression, survival	[5,6,10,12]
**Nanomedicine**	Biomimetic nanoparticles, miRNA delivery	Drug delivery, immune modulation	Splenic, orthotopic	Reduced metastatic burden	[7,10,11]
**Local delivery systems**	Hydrogels, nanovaccines	Sustained local therapy	Orthotopic, liver mets	Local control, immune memory	[8,9,25]
**TME modulation**	STING, CAFs, ferroptosis	Immune reprogramming	Liver mets	Enhanced ICI response	[5,12,26,27,28]
**Metabolic & microbiome targeting**	NAFLD modulation, bile acids, probiotics	Metabolic-immune crosstalk	Liver mets	Reduced metastasis	[4,15,16,20,21]
**Natural products & stromal targeting**	Phytochemicals, EV–CAF, HSC targeting	Stromal disruption	Splenic, orthotopic	Metastasis suppression	[17,18,19,22,23]

**Note:** CRC, colorectal cancer; CRLM, colorectal cancer liver metastasis; 5-FU, 5-fluorouracil; FOLFOX, folinic acid, fluorouracil, and oxaliplatin; TME, tumor microenvironment; VEGF, vascular endothelial growth factor; EGFR, epidermal growth factor receptor.

### 3.1 Immunotherapy and Targeted Combination Strategies

Immune checkpoint blockade has demonstrated limited efficacy in microsatellite-stable colorectal cancer, particularly in the setting of liver metastasis. Preclinical responsiveness to PD-1 blockade is therefore highly model-dependent and frequently observed in immune-competent or combination settings, which may not fully reflect the immunologically ‘cold’ phenotype of most human MSS CRLM. Consequently, combination strategies designed to sensitize CRLM to immunotherapy have been actively explored in preclinical models. Personalized neoantigen vaccination combined with molecular targeted therapy represents one such approach. In a microsatellite-stable CRLM model, the combination of a personalized neoantigen vaccine with regorafenib significantly enhanced CD8^+^ T-cell infiltration, promoted immune memory formation, and remodeled the tumor microenvironment toward an inflamed phenotype, resulting in durable tumor regression and prolonged survival [6].

Beyond vaccination strategies, multiple studies have focused on overcoming intrinsic resistance to immune checkpoint inhibitors. Engineered nanodrugs and biomimetic delivery systems have been shown to sensitize immune-desert liver metastases to anti–PD-L1 therapy by modulating tumor cell signaling and restoring antitumor immunity [10]. Innate immune activation has also emerged as a critical determinant of immunotherapy responsiveness. Activation of macrophage STING signaling enhanced natural killer cell–mediated antitumor immunity through 4-1BBL/4-1BB co-stimulation, leading to effective suppression of liver metastatic burden [12]. Targeting immunosuppressive macrophage pathways further augmented the efficacy of anti–PD-1 therapy by remodeling the hepatic immune microenvironment, underscoring the importance of myeloid cell–focused interventions in CRLM [5].

Nevertheless, it is important to recognize that many immunotherapeutic combinations demonstrating robust tumor regression in murine CRLM models remain at the mechanistic or early translational stage. Differences in immune tolerance, tumor burden heterogeneity, and prior treatment exposure between experimental systems and patients may substantially influence therapeutic durability. Therefore, these findings should be interpreted as hypothesis-generating rather than definitive predictors of clinical efficacy.

### 3.2 Nanomedicine and Local Drug Delivery Systems

Nanomedicine-based platforms have been extensively investigated for their ability to enhance drug delivery, improve pharmacokinetics, and modulate the tumor microenvironment in CRLM. Biomimetic nanoparticles designed to mimic immune cells or cellular membranes have demonstrated enhanced accumulation within liver metastases and improved therapeutic efficacy when combined with chemotherapy or immunotherapy [7,10]. Nanoparticle-mediated delivery of nucleic acids, including microRNAs, has further enabled targeted suppression of metastatic pathways and reduced hepatic tumor burden [11].

Local drug delivery strategies, particularly those employing injectable or implantable hydrogels, have expanded the therapeutic landscape by enabling sustained and localized drug release within orthotopic and metastatic lesions. Thermosensitive and drug-eluting hydrogels have been successfully used to suppress both primary colorectal tumors and liver metastases while minimizing systemic toxicity [8,9]. These biomaterial-assisted systems not only enhance therapeutic efficacy but also improve experimental reproducibility by stabilizing tumor architecture and local drug exposure.

In addition, multifunctional nanoplatforms combining physical or chemical modalities—such as sonodynamic therapy or immune stimulation—have demonstrated synergistic antitumor effects in CRLM models [13,25]. More recently, immune-activating nanovesicles loaded with Toll-like receptor agonists have been shown to reprogram the hepatic immune microenvironment and suppress metastatic growth, highlighting the versatility of nanomedicine-based interventions [30].

Recent advances in immune-engineered nanomedicine have also suggested that modulation of innate immune signaling and macrophage efferocytic function may represent a useful design principle for future nanoparticle-based strategies, although direct validation in CRLM remains limited [47].

However, nanoparticle accumulation and therapeutic magnitude observed in preclinical CRLM models may not fully recapitulate the pharmacokinetic complexity, hepatic clearance mechanisms, and immune sequestration present in patients [48]. Translation of nanomedicine-based strategies therefore requires cautious interpretation, particularly with respect to dosing scalability and long-term safety [49].

Success in mechanistic or model-based preclinical studies does not guarantee translational relevance; many promising preclinical findings fail to advance to clinical success without further rigorous validation, reproducibility checks, and alignment with clinical endpoints [50].

Although nanomedicine-based strategies demonstrate compelling mechanistic effects in controlled experimental systems, many remain at an exploratory or early translational stage, and their clinical scalability requires rigorous cross-platform validation [51].

### 3.3 Tumor Microenvironment and Immune Modulation

The hepatic metastatic niche is characterized by a complex tumor microenvironment (TME) composed of immune cells, stromal elements, and extracellular matrix components that collectively regulate tumor progression and therapeutic response. This subsection primarily focuses on the cellular and stromal components of the tumor microenvironment, including immune cell subsets, cancer-associated fibroblasts, and myeloid-mediated immune suppression, which directly influence therapeutic responsiveness in CRLM. Macrophages, myeloid-derived suppressor cells (MDSCs), and cancer-associated fibroblasts (CAFs) play central roles in establishing immune tolerance and promoting metastatic outgrowth in CRLM.

Several studies have demonstrated that targeting macrophage-mediated immunosuppression can restore antitumor immunity and enhance responsiveness to immune checkpoint blockade [5,12]. Disease-associated conditions such as non-alcoholic fatty liver disease further exacerbate immune suppression by recruiting MDSCs and impairing T-cell function, thereby facilitating metastatic progression and immunotherapy resistance [4]. Remodeling the hepatic immune microenvironment to dismantle T-cell exclusion zones has been shown to markedly improve immunotherapeutic efficacy in liver metastasis models [26].

At the mechanistic level, diverse pathways—including STING signaling, neutrophil extracellular trap (NET) formation, and regulated cell death processes such as ferroptosis—have been implicated in modulating metastatic fitness and therapeutic sensitivity [12,28,29]. Targeting stromal and perivascular components, including CAFs and pericytes, has further revealed previously underappreciated mechanisms driving immune evasion and metastatic colonization [30,31].

### 3.4 Systemic Metabolic and Microbiome-Mediated Immune Modulation

Metabolic regulation and gut–liver axis signaling have emerged as critical modulators of CRLM progression and therapeutic response. In contrast to the cellular emphasis of Section 3.3, this subsection examines soluble metabolic regulators, bile acid signaling, and microbiome-driven immune modulation as systemic determinants of metastatic fitness. Hepatic metabolic states, including dysregulated lipid metabolism and cholesterol synthesis, directly influence immune cell function and metastatic niche formation. Inhibition of cholesterol synthesis within the metastatic niche has been shown to suppress liver metastasis and attenuate tumor growth in preclinical models [16].

Alterations in bile acid metabolism represent another key axis linking gut microbiota to hepatic immune regulation. Microbiome-driven modulation of bile acid composition has been demonstrated to influence metastatic colonization and immune responses in the liver [21]. Consistent with this concept, probiotic supplementation and non-absorbable antibiotic treatment have been shown to reduce metastatic burden by reshaping the intestinal microbiome and downstream hepatic immune signaling [20,21].

Furthermore, metabolic interventions have been shown to modulate immune checkpoint resistance. Pharmacologic targeting of metabolic pathways, including mTOR signaling, restored sensitivity to anti–PD-1 therapy by optimizing the tumor microenvironment and reducing immune exhaustion [15]. Collectively, these findings underscore the importance of metabolic and microbiome-driven mechanisms in shaping therapeutic vulnerability in CRLM.

### 3.5 Natural Products and Stromal-Targeted Therapies

Natural products and traditional medicinal compounds have provided valuable insights into stromal–tumor interactions and microenvironment-focused therapeutic strategies in CRLM. These approaches are discussed within the broader context of tumor microenvironment–targeted strategies, as their primary mechanistic contribution lies in stromal and immune modulation rather than isolated cytotoxic effects. Multiple phytochemicals have demonstrated the ability to suppress liver metastasis by modulating hepatic immune responses and inhibiting stromal cell activation [17,18]. These compounds often exert pleiotropic effects, simultaneously targeting immune cells, extracellular matrix remodeling, and inflammatory signaling pathways.

Targeting the extracellular vesicle (EV)–mediated communication between tumor cells and stromal components has emerged as a particularly promising strategy. Inhibition of EV-driven activation of cancer-associated fibroblasts significantly reduced metastatic progression, highlighting the functional importance of intercellular signaling in the hepatic metastatic niche [19,22]. Similarly, therapeutic approaches targeting hepatic stellate cells have been shown to disrupt stromal support for metastatic growth and attenuate liver colonization [18,23].

Together, these studies illustrate that natural products and stromal-targeted therapies can complement conventional and immune-based treatments by reshaping the hepatic microenvironment and limiting metastatic progression.

### 3.6 Model-Dependent Therapeutic Response Patterns

Across therapeutic categories, a consistent pattern emerges: observed efficacy is strongly influenced by the biological constraints embedded within each experimental platform. Immunotherapy combinations evaluated in immune-competent orthotopic models frequently demonstrate enhanced CD8^+^ T-cell infiltration and durable tumor regression; however, these responses are often attenuated in clinical CRLM, where immune tolerance and myeloid dominance are more pronounced. Conversely, nanomedicine-based delivery systems may show improved tumor accumulation in ectopic models but encounter hepatic clearance barriers and Kupffer cell uptake in orthotopic settings.

Similarly, liver-directed therapies such as ablation and radioembolization induce measurable immune activation in preclinical models, yet clinical translation often reveals discordance between biomarker modulation and overall survival benefit. These observations underscore that therapeutic magnitude, durability, and systemic impact are model-contingent phenomena. Therefore, cross-model comparison is essential before extrapolating preclinical efficacy toward translational expectations.

Importantly, immune-competent orthotopic models more closely reflect the immune-refractory phenotype observed in microsatellite-stable CRLM patients, whereas highly synchronized splenic injection models may overestimate therapeutic magnitude due to reduced metastatic heterogeneity. Consequently, interpretation of preclinical efficacy should be explicitly contextualized according to model-specific biological constraints when drawing parallels to clinical trial outcomes.

In addition, the accelerated kinetics of metastatic establishment in murine models—often occurring within weeks—may incompletely capture the chronic immune exhaustion and evolutionary dynamics that develop over years in human CRLM, thereby potentially overestimating the durability of immunotherapeutic responses.

Notably, reproducibility across model platforms remains variable. Strategies demonstrating robust tumor regression in synchronized splenic injection models may show attenuated effects in orthotopic or immune-competent systems, suggesting that therapeutic magnitude is not inherently generalizable. Comparative evaluation across models is therefore essential to distinguish biologically robust interventions from context-dependent phenomena.

Furthermore, dose–response relationships are inconsistently reported in CRLM preclinical studies. In several cases, efficacy is demonstrated at a single dosing schedule without systematic evaluation of therapeutic thresholds or toxicity margins. Such limitations constrain translational interpretation and underscore the need for standardized evaluative criteria.

Negative or discordant findings—particularly where immune activation does not translate into survival benefit—provide equally important insights into therapeutic durability. Incorporating recurrence modeling and longitudinal survival analysis may therefore improve the predictive validity of preclinical therapeutic evaluation.

## 4 Local and Liver-Directed Therapies in Colorectal Cancer Liver Metastasis

**Figure 4:**
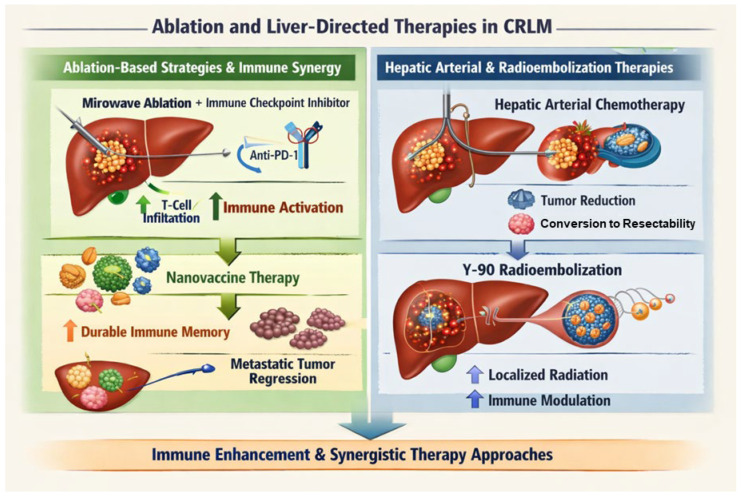
**Local and liver-directed therapeutic strategies for colorectal cancer liver metastasis and their immunological implications.** This schematic illustrates major local and liver-directed therapeutic modalities applied in the treatment of colorectal cancer liver metastasis (CRLM) and highlights their immunological and translational relevance. Thermal ablation strategies, including radiofrequency ablation (RFA) and microwave ablation (MWA), induce localized tumor destruction and promote immunogenic cell death, leading to the release of tumor antigens and activation of antitumor immune responses. These ablation-induced immune effects provide a biological rationale for combination with immunotherapies. Hepatic arterial–based therapies exploit the preferential arterial blood supply of liver metastases to deliver high local concentrations of cytotoxic agents while limiting systemic exposure. In parallel, yttrium-90 (Y-90) radioembolization delivers selective internal radiation to metastatic lesions, achieving intrahepatic tumor control while inducing systemic and local immune modulation. Together, these liver-directed approaches function not only as locoregional cytoreductive therapies but also as immune-modulating interventions that can synergize with systemic chemotherapy and immunotherapy. This figure summarizes how local treatment strategies bridge preclinical mechanistic insights and clinical application in the management of CRLM. In Fig. 4, the upward arrows denote enhanced therapeutic and immunological effects, whereas the connecting arrows illustrate the conceptual relationship between liver-directed interventions and broader immune-enhancing synergistic treatment strategies. A schematic comparison inset illustrates the transition from an immune “cold” metastatic niche characterized by sparse cytotoxic T-cell infiltration and dominant myeloid suppression to an immune “hot” phenotype following ablation or radioembolization, marked by increased CD8^+^ T-cell density and enhanced immune activation. The figure was created using BioRender (BioRender Inc., Toronto, ON, Canada) and assembled in Microsoft PowerPoint (Microsoft 365, Microsoft Corporation, Redmond, WA, USA).

Local and liver-directed therapies play a pivotal role in the management of colorectal cancer liver metastasis (CRLM), particularly in patients with liver-dominant disease or limited systemic treatment options. Beyond their capacity to achieve local tumor control, these interventions exert profound effects on the hepatic immune microenvironment, thereby creating opportunities for synergistic integration with systemic and immune-based therapies. Preclinical studies have provided critical mechanistic insights into ablation-induced immune activation and regional treatment–mediated modulation of antitumor immunity, which are increasingly being translated into clinical practice. (Fig. 4) The mechanisms, immune effects, and clinical implications of liver-directed therapies are summarized in Table 3.

**Table 3:** Local and liver-directed therapies for colorectal cancer liver metastasis: mechanisms, immune effects, and clinical implications.

Therapy Type	Modality	Primary Mechanism	Immune Modulation	Clinical Context	Key References
**Thermal ablation**	RFA	Local tumor necrosis	Antigen release, immune priming	Oligometastatic CRLM	[24]
**Thermal ablation**	MWA	Rapid tumor destruction	CXCL9-mediated T-cell recruitment	Combination with ICI	[24]
**Peri-ablation immunotherapy**	Nanovaccine	Immune memory induction	Systemic antitumor immunity	Adjuvant setting	[25]
**Hepatic arterial therapy**	HA chemotherapy	High intrahepatic drug delivery	Indirect immune modulation	Unresectable CRLM	[35]
**Radioembolization**	Y-90 TARE/SIRT	Internal radiation	Systemic immune changes	Liver-dominant disease	[38,39,45]

**Note:** CRLM, colorectal cancer liver metastasis; MWA, microwave ablation; RFA, radiofrequency ablation; TARE, transarterial radioembolization; HA, Hepatic Arterial; SIRT, Selective Internal Radiation Therapy.

### 4.1 Ablation-Based Strategies and Immune Synergy

Thermal ablation techniques, including radiofrequency ablation (RFA) and microwave ablation (MWA), are widely used for local control of liver metastases and serve as important alternatives or adjuncts to surgical resection [35,36,37]. In addition to direct cytotoxic effects, accumulating evidence indicates that ablation induces immunogenic tumor cell death, leading to the release of tumor-associated antigens and danger-associated molecular patterns that can stimulate systemic antitumor immunity [26,27].

These immunogenic effects have been consistently observed in both experimental metastatic models and early translational studies. Preclinical models of CRLM have demonstrated that ablation-based therapies can synergize with immunomodulatory approaches. In particular, the combination of microwave ablation with immune checkpoint blockade has been shown to enhance intratumoral T-cell infiltration, promote CXCL9-mediated immune recruitment, and suppress both treated and distant metastatic lesions [24]. Similarly, nanovaccine-based strategies applied in the peri-ablation setting have elicited durable immune memory and reduced postoperative recurrence, highlighting the potential of ablation-induced immune priming for long-term disease control [25] (Fig. 4 left panel).

These findings underscore the concept of ablation-induced immunity, whereby local tumor destruction not only achieves immediate cytoreduction but also converts immunologically “cold” metastatic lesions into inflamed, immune-responsive sites. Importantly, the magnitude and durability of immune activation appear to be highly dependent on treatment timing, ablation modality, and combination with systemic immunotherapies, emphasizing the need for optimized treatment sequencing informed by preclinical modeling.

Importantly, ablation-induced immune modulation is context-dependent and may represent a double-edged phenomenon, wherein early immunogenic cell death and antigen release are counterbalanced by compensatory inflammatory or myeloid-mediated immunosuppressive responses; therefore, quantitative immune profiling and rational sequencing with systemic immunotherapy are critical for therapeutic exploitation.

### 4.2 Hepatic Arterial and Radioembolization Therapies

Hepatic arterial therapies exploit the preferential arterial blood supply of liver metastases to deliver high local concentrations of therapeutic agents while minimizing systemic exposure. Hepatic arterial chemotherapy has been investigated as a strategy to enhance intrahepatic tumor control, particularly in patients with unresectable or liver-limited disease. Clinical studies combining hepatic arterial oxaliplatin infusion with systemic chemotherapy and targeted agents have demonstrated encouraging response rates and conversion to resectability in selected patient populations [35].

Radioembolization using yttrium-90 (Y-90) microspheres represents another liver-directed modality that delivers high-dose internal radiation selectively to metastatic lesions. While large, randomized trials have reported limited overall survival benefit when Y-90 radioembolization is combined with first-line systemic chemotherapy, these studies have nonetheless demonstrated improved hepatic disease control and provided critical insights into patient selection and treatment timing [45]. Importantly, emerging evidence indicates that Y-90 radioembolization induces systemic and intrahepatic immune modulation, including changes in circulating immune cell populations and immune-related biomarkers [38,39].

Recent prospective and observational studies have further characterized the immunological consequences of Y-90 radioembolization, revealing alterations in T-cell activation, cytokine profiles, and immune checkpoint expression in both treated and untreated hepatic lesions [38,39]. These findings suggest that radioembolization may function as an immune-modulating therapy rather than a purely cytotoxic intervention, thereby providing a biological rationale for combination strategies incorporating immunotherapy or immune-priming agents.

Collectively, hepatic arterial chemotherapy and Y-90 radioembolization highlight the evolving role of liver-directed therapies as both locoregional control measures and immunologically active interventions. Preclinical and translational studies will be essential to define optimal patient selection, treatment sequencing, and combination regimens that maximize both local and systemic therapeutic benefits. (Fig. 4 Right panel) The top arrow represents the transition from local tumor ablation to systemic immune activation.

## 5 Translational and Clinical Perspectives

**Figure 5:**
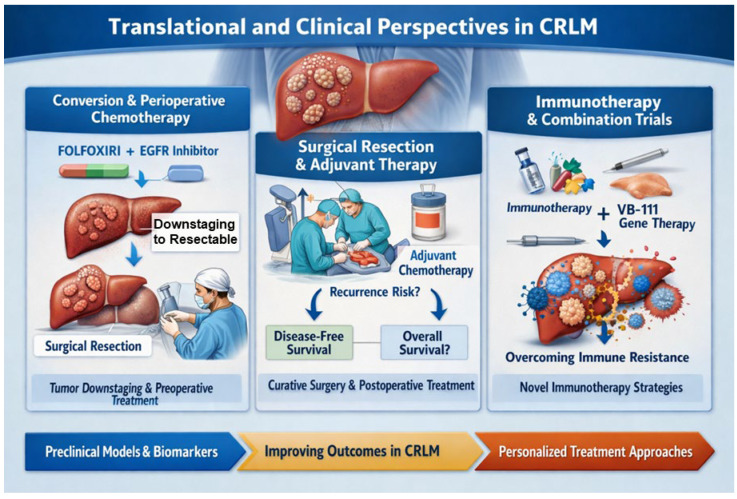
**Translational and clinical perspectives in the management of colorectal cancer liver metastasis (CRLM).** This figure summarizes the translational and clinical strategies for colorectal cancer liver metastasis (CRLM), highlighting how insights from preclinical models inform contemporary clinical decision-making and personalized treatment approaches. The left panel illustrates conversion and perioperative chemotherapy strategies, in which intensified systemic regimens, such as FOLFOXIRI combined with epidermal growth factor receptor (EGFR) inhibitors, are used to downstage initially unresectable liver metastases and enable curative-intent surgical resection. The central panel depicts surgical resection and adjuvant therapy as the cornerstone of CRLM management. Hepatectomy followed by adjuvant chemotherapy aims to improve disease-free survival; however, the risk of recurrence and its impact on overall survival remain key clinical challenges. The right panel highlights emerging immunotherapy and combination clinical trials designed to overcome immune resistance in microsatellite-stable CRLM. Combination approaches, including immune checkpoint blockade with tumor-targeting gene therapy (e.g., VB-111), illustrate novel strategies to enhance antitumor immunity within the hepatic metastatic microenvironment. The lower schematic emphasizes the integrative role of preclinical models and biomarker-driven assessment in guiding translational research, improving clinical outcomes, and advancing personalized treatment strategies for patients with CRLM. The figure was created using BioRender (BioRender Inc., Toronto, ON, Canada) and assembled in Microsoft PowerPoint (Microsoft 365, Microsoft Corporation, Redmond, WA, USA).

The clinical management of colorectal cancer liver metastasis (CRLM) has evolved substantially over the past two decades, driven by advances in systemic therapy, liver-directed interventions, and surgical techniques. Importantly, many of these developments have been informed—directly or indirectly—by insights gained from preclinical models that capture the biological complexity of hepatic metastasis. This section reviews key translational and clinical strategies for CRLM, focusing on conversion and perioperative chemotherapy, surgical resection with adjuvant treatment, and emerging immunotherapy-based combination trials (Fig. 5).

Importantly, clinical observations discussed in this section are re-examined through the same evaluative framework applied to preclinical models, allowing reverse-translational interpretation of discrepancies between radiographic response, immune modulation, and long-term survival outcomes.

Beyond conceptual alignment between preclinical findings and clinical observations, an operational translational framework is required to bridge experimental insight with actionable clinical decision-making. Specifically, preclinical CRLM models should inform: (i) patient selection based on immune contexture, metabolic status, and metastatic burden; (ii) biomarker identification and validation, including imaging-derived heterogeneity metrics and immune signatures; and (iii) rational treatment sequencing, particularly the integration of liver-directed therapies with systemic immunotherapy. Immune-competent orthotopic models, for example, may help define biological thresholds for immune exclusion and myeloid dominance that correspond to microsatellite-stable, immunotherapy-resistant patient subsets. Similarly, synchronized splenic injection models may inform early colonization dynamics relevant to minimal residual disease or perioperative intervention timing. By explicitly mapping model-dependent biological features to clinical trial design and stratification strategies, translational interpretation becomes actionable rather than conceptual.

### 5.1 Conversion and Perioperative Chemotherapy Strategies

Systemic chemotherapy remains a cornerstone of CRLM management, particularly in patients with initially unresectable or borderline resectable disease. Conversion therapy aims to downstage liver metastases to enable curative-intent surgical resection. The CAIRO5 randomized clinical trial demonstrated that intensified systemic regimens incorporating FOLFOXIRI or FOLFOX combined with targeted agents could achieve meaningful conversion to resectability and improve progression-free survival in selected patients with liver-limited metastatic disease [1]. These findings underscore the importance of aggressive systemic control in the preoperative setting.

The integration of epidermal growth factor receptor (EGFR) inhibitors into chemotherapy backbones has further enhanced tumor response rates in molecularly selected populations. A randomized controlled trial comparing cetuximab plus FOLFOXIRI versus cetuximab plus FOLFOX showed superior objective response rates and conversion outcomes with intensified combination therapy, albeit at the cost of increased toxicity [32]. Earlier landmark studies, including the CELIM trial, established the feasibility of EGFR-targeted therapy combined with chemotherapy for downstaging unresectable CRLM, enabling secondary resection in a substantial proportion of patients [44].

However, long-term outcomes following perioperative chemotherapy have proven heterogeneous. The EORTC 40983 trial demonstrated improved progression-free survival with perioperative FOLFOX4 chemotherapy compared with surgery alone, but failed to show a significant overall survival benefit with extended follow-up [42,43]. Similarly, the New EPOC trial reported inferior survival outcomes when cetuximab was added to perioperative chemotherapy in patients with resectable CRLM, highlighting the complexity of treatment selection and the potential for adverse interactions between targeted agents and surgical timing [43]. Collectively, these studies emphasize that response rate alone may be an insufficient surrogate for long-term benefit and that biologically informed patient stratification remains essential.

These clinical discrepancies underscore the need for preclinical models that incorporate endpoints beyond tumor shrinkage, including recurrence modeling, immune durability, and metastatic heterogeneity. Preclinical platforms that fail to capture these dimensions may misinform trial design by overemphasizing radiographic response rather than long-term survival benefit.

These discrepancies further highlight that therapeutic evaluation should incorporate comparative and longitudinal endpoints, rather than relying solely on short-term tumor regression metrics.

From a reverse-translational perspective, these clinical observations highlight limitations in preclinical modeling paradigms. Many preclinical platforms prioritize tumor shrinkage as a primary endpoint, whereas clinical outcomes depend on durable immune control, prevention of micrometastatic recurrence, and long-term survival. The discordance between radiographic response and overall survival observed in trials such as New EPOC and EORTC 40983 suggests that preclinical therapeutic evaluation should incorporate endpoints that better approximate clinical durability, including recurrence modeling and longitudinal immune monitoring.

### 5.2 Surgical Resection and Adjuvant Treatment

Surgical resection remains the only potentially curative treatment for CRLM and is central to long-term disease control [42,43]. Advances in surgical technique, perioperative management, and patient selection have expanded the pool of patients eligible for hepatectomy. Nevertheless, recurrence rates following curative-intent resection remain high, prompting continued investigation into the role of adjuvant systemic therapy.

Randomized trials evaluating adjuvant chemotherapy after hepatectomy have yielded mixed results. The JCOG0603 trial demonstrated improved disease-free survival with adjuvant mFOLFOX6 following hepatic resection, but failed to show a corresponding overall survival benefit, raising questions regarding the clinical significance of intensified postoperative treatment [33]. Other studies comparing surgery alone with perioperative or postoperative chemotherapy have similarly reported discordant effects on recurrence and survival, reflecting the biological heterogeneity of CRLM and the influence of micro metastatic disease [2,34,42].

These findings suggest that while systemic therapy can reduce early recurrence, its impact on long-term survival may be limited by underlying tumor biology and metastatic potential. Increasing attention has therefore been directed toward identifying biomarkers and imaging-based predictors that can guide adjuvant treatment decisions and avoid overtreatment in patients with favorable disease biology.

To operationalize biomarker-driven adjuvant strategies, preclinical models should incorporate recurrence modeling and longitudinal immune profiling that parallel postoperative surveillance settings. For instance, immune-competent orthotopic models that simulate micrometastatic persistence may provide a platform for validating circulating biomarkers, radiomic signatures, or immune activation thresholds predictive of recurrence risk. Such alignment would enable biomarker development pipelines that directly inform postoperative treatment stratification.

### 5.3 Immunotherapy and Combination Clinical Trials

Despite transformative success in other malignancies, immune checkpoint inhibitors have shown limited efficacy in the majority of patients with microsatellite-stable CRLM. Consequently, recent clinical efforts have focused on combination strategies designed to overcome immune resistance within the hepatic metastatic microenvironment.

One notable example is the phase II trial evaluating VB-111 (ofranergene obadenovec), a tumor-targeting gene therapy, in combination with nivolumab in patients with microsatellite-stable CRLM. This study demonstrated manageable safety and evidence of immune activation, with a subset of patients exhibiting durable disease control and immune biomarker modulation [36]. Although objective response rates remained modest, these findings provide proof-of-concept that rationally designed combination strategies can partially overcome immune exclusion in liver metastases.

The modest magnitude of clinical response may reflect limitations of current preclinical platforms in recapitulating chronic immune exhaustion, spatial heterogeneity, and myeloid-dominant immunosuppression characteristic of advanced MSS CRLM. While immune activation was evident in experimental systems, these models may incompletely represent long-term hepatic immune tolerance mechanisms present in patients.

Importantly, these clinical observations mirror preclinical findings demonstrating that immune modulation in the liver—through vascular targeting, stromal reprogramming, or local immune priming—can sensitize metastatic lesions to immunotherapy. Ongoing and future trials integrating immunotherapy with liver-directed modalities, such as ablation or radioembolization, are expected to further refine combination strategies and identify patient populations most likely to benefit. Key clinical trials and translational strategies in CRLM are summarized in Table 4.

Treatment sequencing should be biologically informed. Preclinical evidence indicating that ablation or radioembolization induces transient immune activation suggests a defined therapeutic window for subsequent immune checkpoint blockade. Clinical translation therefore requires temporal mapping of immune activation kinetics observed in preclinical models to guide optimal scheduling in combination trials. Without such mechanistic sequencing alignment, synergistic potential may remain unrealized.

**Table 4:** Key clinical trials and translational strategies in colorectal cancer liver metastasis.

Strategy Category	Trial/Study	Treatment Regimen	Patient Population	Key Outcomes	References
**Conversion therapy**	CAIRO5	FOLFOX/FOLFOXIRI ± targeted agents	Initially unresectable CRLM	Conversion to resection, PFS	[1]
**Intensified chemotherapy**	Cetuximab + FOLFOXIRI vs. FOLFOX	Chemotherapy ± EGFR inhibitor	Liver-limited CRLM	ORR, conversion	[32]
**Perioperative therapy**	EORTC 40983	Surgery ± perioperative FOLFOX4	Resectable CRLM	PFS↑, OS NS	[2,42]
**EGFR perioperative**	New EPOC	Chemotherapy ± cetuximab	Resectable CRLM	OS↓ with EGFR	[43]
**Adjuvant therapy**	JCOG0603	Hepatectomy ± mFOLFOX6	Liver-only metastasis	DFS↑, OS NS	[33]
**Immunotherapy combo**	VB-111 + nivolumab	Gene therapy + ICI	MSS CRLM	Immune activation, DCR	[36]

**Note:** PFS, progression-free survival; OS, overall survival; DFS, disease-free survival; ORR, objective response rate; DCR, disease control rate; MSS, microsatellite stable; ICI, immune checkpoint inhibitor. ↑ indicates improvement; ↓ indicates deterioration; NS indicates not statistically significant.

## 6 Precision Imaging and Response Assessment

Accurate assessment of therapeutic response and disease heterogeneity is essential for optimizing treatment strategies in colorectal cancer liver metastasis (CRLM). Conventional response criteria based on lesion size reduction often fail to capture early biological changes, spatial heterogeneity, and microenvironmental remodeling induced by modern systemic and liver-directed therapies. Advances in precision imaging, including radiomics and molecular imaging, have therefore emerged as critical tools for noninvasive response assessment, treatment stratification, and translational bridging between preclinical models and clinical practice.

### 6.1 Radiomics and Imaging-Based Outcome Prediction

Radiomics-based approaches extract high-dimensional quantitative features from routine imaging modalities, such as computed tomography (CT) and magnetic resonance imaging (MRI), to characterize tumor phenotype beyond visual assessment. In the context of CRLM, radiomics has demonstrated considerable potential for predicting therapeutic response, progression-free survival, and overall survival following liver-directed therapies.

Recent studies have shown that radiomics-derived signatures can stratify patients undergoing yttrium-90 (Y-90) radioembolization according to treatment response and clinical outcome. By integrating texture, shape, and intensity features, radiomics-based prediction models have outperformed conventional imaging criteria in identifying patients likely to benefit from radioembolization [40]. Similarly, imaging biomarkers derived from post-treatment scans have been shown to capture early response dynamics and systemic immunological effects following liver-directed interventions [41].

Importantly, radiomics provides a noninvasive means to assess intra-patient and inter-lesional heterogeneity, a key determinant of therapeutic resistance in CRLM. These imaging-based features may serve as surrogate markers of underlying biological processes, including vascularity, fibrosis, immune infiltration, and metabolic activity. As such, radiomics represents a promising platform for treatment stratification and adaptive therapy design, particularly when integrated with clinical and molecular data.

### 6.2 Molecular Imaging and Target Heterogeneity

While radiomics captures phenotypic heterogeneity at the macroscopic level, molecular imaging enables direct visualization of target expression and biological processes *in vivo*. Immuno–positron emission tomography (immuno-PET) has emerged as a powerful modality for assessing target distribution, pharmacokinetics, and heterogeneity across metastatic lesions.

First-in-human studies using site-specifically labeled immuno-PET tracers have demonstrated the feasibility of noninvasively evaluating receptor expression and spatial heterogeneity in metastatic disease. In particular, immuno-PET imaging with zirconium-89–labeled monoclonal antibodies has revealed substantial interlesional and intralesional heterogeneity in HER2 expression, which cannot be reliably inferred from a single-site biopsy [46]. Such heterogeneity has direct implications for patient selection, therapeutic targeting, and resistance to antibody-based therapies.

In CRLM, where metastatic lesions frequently exhibit divergent biological profiles within the same patient, molecular imaging provides a critical advantage by enabling whole-body assessment of target expression. This capability is particularly relevant for guiding precision therapy, monitoring treatment engagement, and identifying discordant lesions that may drive disease progression. Integration of molecular imaging with preclinical model–derived biomarkers further enhances translational relevance, enabling iterative refinement of therapeutic strategies based on real-time biological readouts.

**Figure 6:**
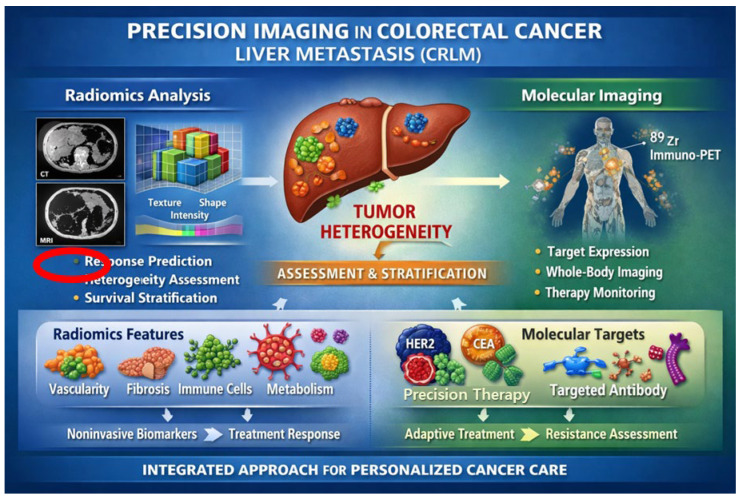
**Precision imaging approaches for assessment of tumor heterogeneity and therapeutic response in colorectal cancer liver metastasis (CRLM).** This figure illustrates integrated precision imaging strategies for noninvasive assessment of tumor heterogeneity and therapeutic response in colorectal cancer liver metastasis (CRLM). The left panel depicts radiomics analysis based on computed tomography (CT) and magnetic resonance imaging (MRI), in which quantitative features related to texture, shape, and intensity are extracted to enable response prediction, heterogeneity assessment, and survival stratification. Radiomics-derived features reflect underlying biological processes, including tumor vascularity, fibrosis, immune cell infiltration, and metabolic activity, and serve as noninvasive biomarkers of treatment response. The central schematic highlights intrapatient and interlesional tumor heterogeneity as a critical determinant of therapeutic outcome in CRLM, emphasizing the need for comprehensive assessment beyond size-based criteria. The right panel illustrates molecular imaging using immuno–positron emission tomography (immuno-PET) with zirconium-89–labeled antibodies, which enables whole-body visualization of target expression, monitoring of therapy engagement, and assessment of target heterogeneity across metastatic lesions. Molecular imaging supports precision therapy selection and adaptive treatment strategies by identifying discordant or resistant tumor sites. Together, radiomics and molecular imaging provide complementary, noninvasive tools for treatment stratification, response monitoring, and personalized management of colorectal cancer liver metastasis. The figure was created using BioRender (BioRender Inc., Toronto, ON, Canada) and assembled in Microsoft PowerPoint (Microsoft 365, Microsoft Corporation, Redmond, WA, USA).

To close the translational loop, imaging-derived heterogeneity metrics should not be viewed solely as descriptive tools but as candidate predictive biomarkers. Integrating radiomic signatures and molecular imaging parameters with model-derived immune and stromal profiles may enable the construction of clinically deployable composite biomarkers. Such integrative pipelines can guide patient selection for liver-directed therapies, immunotherapy combinations, or intensified perioperative regimens based on measurable biological risk stratification. A schematic overview of precision imaging-guided therapeutic stratification in CRLM is presented in Fig. 6. Precision imaging approaches for response assessment and treatment stratification are summarized in Table 5.

**Table 5:** Precision imaging approaches for response assessment and treatment stratification in colorectal cancer liver metastasis.

Imaging Modality	Approach	Key Information Provided	Clinical Utility	Representative References
**CT/MRI radiomics**	Feature extraction & modeling	Tumor texture, shape, heterogeneity	Response prediction, outcome stratification	[40,41]
**Radiomics (post–liver-directed therapy)**	Longitudinal analysis	Early treatment response	Patient selection for Y-90 or ablation	[40]
**Immuno-PET**	Target-specific antibody imaging	Receptor expression, heterogeneity	Precision targeting, patient selection	[46]
**Molecular imaging**	Whole-body assessment	Interlesional heterogeneity	Resistance monitoring	[46]
**Integrated imaging**	Imaging + biomarkers	Phenotype–biology linkage	Translational research	—

**Note:** CT, computed tomography; MRI, magnetic resonance imaging; PET, positron emission tomography; Y-90, Yittrium-90; “—” indicates that data are not available or that the parameter is not applicable.

## 7 Translational Challenges and Future Directions in CRLM

Despite substantial progress in the development of preclinical models and therapeutic strategies for colorectal cancer liver metastasis (CRLM), significant challenges remain in translating experimental findings into durable clinical benefit. Although thematically linked to precision response assessment discussed in the preceding section, this section broadens the focus to encompass systemic translational challenges, model-dependent bias, and future strategic directions in CRLM research. Accumulating evidence indicates that discrepancies between preclinical efficacy and clinical outcomes are frequently driven by model-dependent bias, incomplete representation of immune and stromal complexity, and inadequate assessment of spatial and temporal heterogeneity [3,5,11,26]. Addressing these limitations will be essential for improving the predictive value of preclinical studies and advancing precision oncology in CRLM.

### 7.1 Impact of Model Choice on Translational Failure

One of the most persistent challenges in CRLM research is the reliance on experimental models that inadequately reflect the biological and therapeutic complexity of liver metastases. Conventional subcutaneous or ectopic tumor models often overestimate therapeutic efficacy by failing to recapitulate the hepatic metastatic niche, immune tolerance, and stromal interactions that govern treatment resistance. As a result, therapies demonstrating robust antitumor activity in ectopic settings have frequently yielded disappointing or inconsistent results in clinical trials.

Such discrepancies do not necessarily invalidate preclinical findings but rather highlight the necessity of aligning experimental model biology with clinical disease complexity. This model-dependent bias has been particularly evident in the context of targeted and immune-based therapies. Clinical trials evaluating perioperative chemotherapy and EGFR-targeted strategies have revealed paradoxical or marginal survival benefits despite strong preclinical rationale, underscoring the limitations of models that do not capture liver-specific immune regulation and microenvironmental constraints. These observations highlight the need to critically reassess how preclinical efficacy is interpreted and to prioritize models that reflect clinically relevant disease biology rather than technical convenience.

Importantly, translational discrepancies should not be interpreted solely as therapeutic failure but often reflect model-dependent bias. Systematic comparison across complementary model platforms, combined with longitudinal and immune-aware endpoints, may help preempt clinical failure by identifying context-dependent therapeutic limitations prior to trial initiation.

### 7.2 Need for Immune-Competent Metastatic Models

The limited success of immunotherapy in microsatellite-stable CRLM has drawn attention to the unique immunological features of the liver metastatic microenvironment. Immune tolerance, myeloid cell–mediated suppression, and dysfunctional antigen presentation collectively limit effective antitumor immunity in hepatic metastases. However, many commonly used preclinical models rely on immunodeficient hosts, precluding meaningful evaluation of immune–tumor interactions and immunotherapy response.

Future progress will require broader adoption of immune-competent metastatic models that enable interrogation of both innate and adaptive immune responses within the liver. Syngeneic orthotopic and metastatic models, particularly those incorporating clinically relevant immune checkpoints and myeloid populations, are critical for evaluating combination strategies designed to overcome immune resistance. Moreover, incorporating disease-associated conditions such as non-alcoholic fatty liver disease or treatment-induced immune remodeling will further enhance the translational relevance of these models.

### 7.3 Spatial Heterogeneity and Longitudinal Assessment

Another major barrier to translational success in CRLM is the underappreciation of spatial and temporal heterogeneity. Liver metastases frequently exhibit substantial interlesional and intralesional variability in molecular signaling, immune infiltration, vascularization, and treatment sensitivity within the same patient [5,26,27]. Single-lesion biopsy or endpoint-based preclinical analyses often fail to capture this complexity, leading to incomplete or misleading conclusions regarding therapeutic efficacy.

Advances in precision imaging, including radiomics and molecular imaging, offer powerful tools for addressing this challenge. Longitudinal imaging enables noninvasive monitoring of treatment response, adaptive resistance, and microenvironmental remodeling over time, providing dynamic insight into therapy-induced biological changes. Integrating spatially resolved imaging data with preclinical models will be essential for identifying resistant subclones, optimizing treatment sequencing, and designing adaptive therapeutic strategies that reflect real-world disease evolution.

### 7.4 Toward Humanized and Patient-Specific Models

To further bridge the translational gap, there is a growing need for humanized and patient-specific modeling platforms. Patient-derived xenografts, organoids, and *ex vivo* culture systems preserve tumor-intrinsic heterogeneity and, when combined with human immune components, offer promising avenues for personalized therapeutic testing. These platforms enable evaluation of drug response in a patient-specific context and may facilitate biomarker discovery and treatment stratification.

Patient-derived organoids and *ex vivo* liver metastasis cultures preserve tumor-intrinsic heterogeneity and enable medium-throughput drug testing. When integrated with humanized immune mouse models and computational multi-omics analysis, these platforms may facilitate patient-specific therapeutic stratification and biomarker validation.

However, humanized models remain technically complex, resource-intensive, and limited by incomplete immune reconstitution. Future efforts should focus on standardizing these systems, improving immune fidelity, and integrating them with orthotopic and metastatic modeling approaches. Combining patient-derived models with longitudinal imaging and multi-omics profiling holds particular promise for creating predictive, individualized treatment pipelines for CRLM.

### 7.5 Future Directions and Translational Outlook

Moving forward, improving outcomes for patients with CRLM will require a paradigm shift in how preclinical models are developed, validated, and applied. Emphasis should be placed on biologically faithful metastatic models, immune competence, and integration of spatial and temporal assessment tools. Rather than seeking universal therapeutic solutions, future research should prioritize model-informed, patient-specific strategies that reflect the heterogeneity and adaptive nature of metastatic disease.

Ultimately, aligning preclinical modeling with clinical complexity—through rational model selection, immune-aware experimental design, and integration of precision imaging—will be essential for overcoming translational barriers and realizing the full potential of emerging therapies for colorectal cancer liver metastasis.

## 8 Conclusions

Colorectal cancer liver metastasis (CRLM) remains a major clinical challenge, driven by its biological complexity, heterogeneous therapeutic responses, and the unique immunological and stromal landscape of the liver. This review has been deliberately structured as a model-informed translational analysis rather than a conventional therapeutic summary, emphasizing that preclinical efficacy must be interpreted within biologically faithful experimental contexts. Although advances in systemic chemotherapy, targeted therapy, immunotherapy, and liver-directed interventions have expanded treatment options, durable clinical benefit remains limited for many patients. These challenges underscore the critical importance of biologically relevant preclinical models capable of accurately predicting therapeutic efficacy and guiding translational development.

In this review, we highlight how orthotopic and metastatic preclinical models have reshaped the evaluation of therapeutic strategies for CRLM by capturing key aspects of hepatic colonization, tumor–microenvironment interactions, and immune regulation. Methodological innovations, including tissue adhesive–based implantation and biomaterial-assisted approaches, have improved reproducibility and experimental precision, enabling more reliable assessment of treatment response. Using these platforms, a broad spectrum of therapeutic strategies—ranging from immunotherapy and targeted combinations to nanomedicine-based delivery systems, metabolic and microbiome modulation, and natural product–based stromal targeting—has been systematically evaluated in preclinical settings.

Importantly, we emphasize that local and liver-directed therapies, such as thermal ablation and radioembolization, function not only as cytoreductive interventions but also as immune-modulating treatments that may synergize with systemic and immune-based therapies. Translational insights from preclinical models have informed clinical strategies, including conversion and perioperative chemotherapy, surgical resection with adjuvant treatment, and emerging immunotherapy-based combination trials. However, discrepancies between preclinical promise and clinical outcomes highlight persistent model-dependent bias and the limitations of experimental systems that fail to recapitulate immune competence, spatial heterogeneity, and disease evolution.

Advances in precision imaging, including radiomics and molecular imaging, provide powerful noninvasive tools for response assessment, treatment stratification, and longitudinal monitoring of tumor heterogeneity. Integration of these imaging modalities with preclinical modeling offers a critical bridge between experimental biology and clinical decision-making, enabling adaptive and personalized therapeutic strategies. It is important to clarify that while these strategies show promising mechanistic results in preclinical models, mechanistic proof-of-concept alone does not necessarily imply readiness for clinical translation, and rigorous validation across diverse model systems is essential before advancing to human studies. Looking forward, the development of immune-competent, humanized, and patient-specific metastatic models—combined with longitudinal imaging and multi-omics profiling—will be essential for overcoming translational barriers and improving predictive accuracy.

A central conclusion of this review is that therapeutic efficacy in CRLM is fundamentally model-contingent. Importantly, preclinical therapeutic success should not be equated with clinical readiness. Robust validation across complementary model systems, durability assessment, and alignment with clinically meaningful endpoints remain essential prerequisites before translational extrapolation. Mechanistic insight, while biologically valuable, represents only an initial stage in translational progression. Robust validation across independent platforms, durability assessment, and alignment with human disease complexity remain prerequisites for credible clinical extrapolation. The predictive value of preclinical findings depends less on the magnitude of tumor regression observed and more on whether the experimental platform faithfully recapitulates immune tolerance, stromal architecture, metabolic constraints, and spatial heterogeneity of hepatic metastasis. Therefore, claims of translational relevance should be interpreted as conditional and hypothesis-generating rather than definitive predictors of clinical efficacy, particularly in the absence of cross-platform validation and longitudinal survival assessment. Future progress in CRLM translational research will require deliberate alignment between model biology and clinical disease complexity, rather than reliance on isolated efficacy signals.

Translational progress will depend not only on biologically faithful modeling but on systematic integration of model-derived biomarkers, patient stratification algorithms, and treatment sequencing principles into prospective clinical trial design.

True therapeutic evaluation in CRLM models requires analytical rigor encompassing reproducibility, cross-model validation, dose-dependency, durability, and alignment with clinically meaningful endpoints. Without these dimensions, preclinical efficacy risks being overinterpreted as translational promise.

Ultimately, improving outcomes for patients with CRLM will require a paradigm shift from one-size-fits-all treatment approaches toward model-informed, biologically grounded, and patient-tailored strategies. By aligning preclinical modeling more closely with clinical complexity, and by embracing immune context, spatial heterogeneity, and dynamic disease evolution, future research can better translate experimental advances into meaningful clinical benefits for patients with colorectal cancer liver metastasis.

## Data Availability

The datasets used and/or analyzed during the current study are available from the corresponding authors on reasonable request.
